# Glucocorticoid‐induced hyperglycaemia in respiratory disease: a systematic review and meta‐analysis

**DOI:** 10.1111/dom.12739

**Published:** 2016-08-04

**Authors:** S. Breakey, S. J. Sharp, A. I. Adler, B. G. Challis

**Affiliations:** ^1^ Department of Medicine, Wolfson Diabetes and Endocrine Clinic, Institute of Metabolic ScienceAddenbrooke's HospitalCambridgeUK; ^2^ MRC Epidemiology Unit, Institute of Metabolic ScienceUniversity of Cambridge School of Clinical MedicineCambridgeUK

**Keywords:** endocrine therapy, glycaemic control, meta‐analysis

## Abstract

The relative risk of glucocorticoid‐induced hyperglycaemia is poorly quantified. We undertook a meta‐analysis to estimate the association between glucocorticoid treatment and hyperglycaemia, overall and separately in individuals with and without diabetes and underlying respiratory disease. We searched electronic databases for clinical trials of adults randomized to either glucocorticoid treatment or placebo. Eight articles comprising 2121 participants were identified. We performed a random effects meta‐analysis to determine relative risks for the associations between glucocorticoid use and both hyperglycaemia and starting hypoglycaemic therapy. In all individuals, the relative risk of hyperglycaemia comparing glucocorticoid treatment with placebo was 1.72 [95% confidence interval (CI) 1.50‐2.04; p < .001]. The relative risks in individuals with and those without diabetes were 2.10 (95% CI 0.92‐5.02; p = .079) and 1.50 (95% CI 0.79‐2.86; p = .22), respectively. In all individuals, the relative risk of hyperglycaemia requiring initiation of hypoglycaemic therapy, comparing glucocorticoid treatment with placebo, was 1.73 (95% CI 1.40‐2.14; p < .001). In conclusion, glucocorticoid therapy increases the risk of hyperglycaemia in all individuals with underlying respiratory disease but not when diabetic status is analysed separately.

## 1 INTRODUCTION

Glucocorticoids are used to treat several diseases because of their anti‐inflammatory and immunosuppressive properties. In the UK, the prevalence of glucocorticoid use at any one time is ~1%.^1^ Among hospital inpatients, prevalence exceeds 10%, with most patients receiving glucocorticoids to treat underlying respiratory illness.^1^ Glucocorticoids are associated with well recognized metabolic complications, including hyperglycaemia. In patients with acute exacerbations of chronic obstructive pulmonary disease (COPD) and community‐acquired pneumonia, hyperglycaemia is associated with prolonged hospital admissions and mortality.^2^

Current data regarding glucocorticoid‐induced hyperglycaemia are based on data from retrospective prescription databases^3^ or prospective studies, which vary in glucocorticoid type, dose, treatment regime, administration route and underlying disease.^4^ These studies show that glucocorticoids worsen hyperglycaemia in people with known diabetes; however, the magnitude of this risk, and the risk of developing hyperglycaemia after glucocorticoid exposure in people without diabetes, is not clear.

Using meta‐analytical techniques, the present study estimates the relative risk of glucocorticoid treatment and hyperglycaemia in individuals with and without diabetes and underlying respiratory disease. It also estimates the association between glucocorticoid use and initiation of hypoglycaemic therapy.

## 2 RESEARCH DESIGN AND METHODS

### 2.1 Literature search

We searched the electronic databases MEDLINE, EMBASE, Cochrane and ClinicalTrials.gov. We searched MEDLINE using standard medical subject heading (MeSH) terms (“glucocorticoids,” “pneumonia,” “pulmonary disease,” “diabetes mellitus” and “community‐acquired infection”) and free text (“respiratory,” “hyperglycaemia,” “steroid” and “prednisolone”) terms. We searched EMBASE using “map term to subject heading” in Advanced Ovid Search. We scanned reference lists of relevant articles to supplement our literature search. We did not restrict the date of study publication.

### 2.2 Study selection

We identified citations in MEDLINE (n = 262), EMBASE (n = 1475), Cochrane Database (n = 138) and ClinicalTrials.gov (n = 112), plus 48 additional citations obtained through scanning reference lists. We included studies with patients aged ≥18 years not treated at baseline with systemic (including inhaled) glucocorticoids for underlying COPD, pneumonia or interstitial lung disease. We included trials if participants received orally or intravenously administered glucocorticoids (≥5 mg prednisolone equivalent) or placebo and/or additional therapy for underlying pulmonary disease for at least 72 hours. We included studies that documented glycaemia throughout, including diabetic status at baseline, and that documented when therapy for hyperglycaemia started. Our primary outcome was hyperglycaemia, as defined by each study‐specific protocol. Our secondary outcome was hyperglycaemia requiring therapy. Eight studies, comprising 2121 participants, met our inclusion criteria (Supplementary Figure S1).

### 2.3 Data abstraction

We collected the following data from each study: study design; year of publication; number of participants; age; sex; underlying respiratory disease; diabetes status at study entry; type and dose of glucocorticoid; method and frequency of quantifying blood glucose; new hyperglycaemia; new hypoglycaemic therapy in response to hyperglycaemia; type of hypoglycaemic agent; and length of follow‐up. Where published data were not available, we communicated with investigators of included studies to obtain additional data about the diabetic status of participants.

### 2.4 Statistical analysis

We calculated a pooled estimate of the association between glucocorticoid treatment and hyperglycaemia by combining the study‐specific relative risks and absolute risk difference using random effects meta‐analysis. We assessed heterogeneity between studies using the I^2^‐statistic. To investigate small study effects, we used Begg's funnel plot.

## 3 RESULTS

### 3.1 Description of studies

Supplementary Table S1 displays the details of the trial characteristics, all of which were placebo‐controlled.^5^, ^6^, ^7^, ^8^, ^9^, ^10^, ^11^, ^12^ The primary endpoints of the trials were respiratory measures rather than hyperglycaemia. All trials were double‐blind with the exception of one open‐label study.^6^ The proportion of participants with pre‐existing diabetes at study entry was provided in seven studies and ranged from 10.3% to 28.9%.

### 3.2 Relative risk of hyperglycaemia after glucocorticoid treatment in all individuals

**Figure 1:**
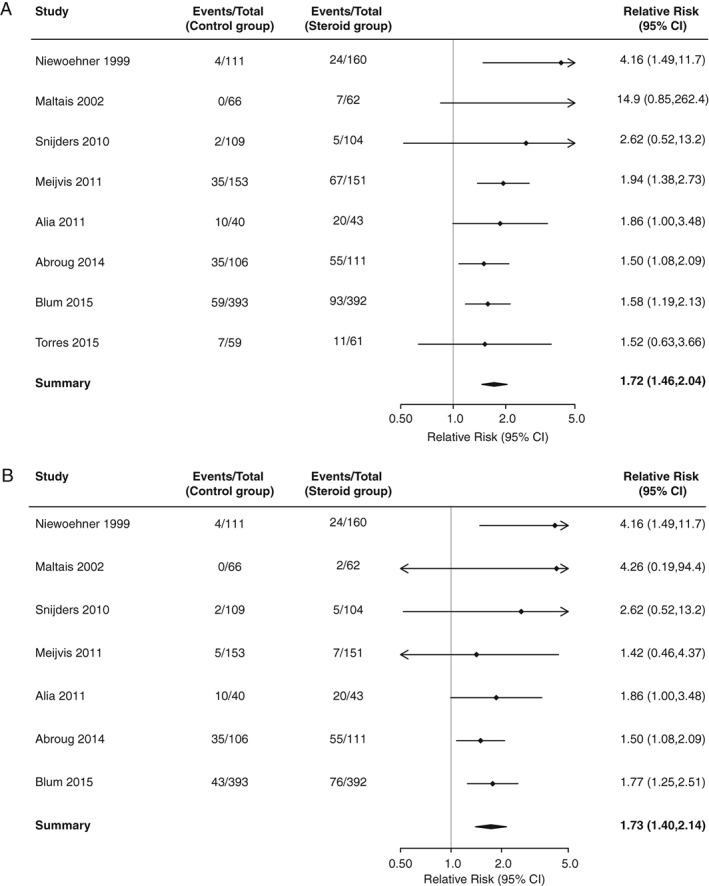
(A) Relative risk of hyperglycaemia, comparing glucocorticoid treatment with placebo in all individuals. (B) Relative risk of hyperglycaemia requiring initiation of hypoglycaemic therapy, comparing glucocorticoid treatment with placebo in all individuals.

Based on eight studies, the relative risk of any hyperglycaemia, comparing glucocorticoid treatment with placebo, was 1.72 [95% confidence interval (CI) 1.50‐2.04; p < .001; I^2^ = 0% (Figure 1A)]. The funnel plot suggests publication bias, with smaller, less precise studies reporting positive associations (Supplementary Figure S2). The number‐needed‐to‐treat for an additional harmful outcome (NNTH) was calculated for the primary outcome. For baseline risks of 5% and 15%, the NNTH was 28 (95% CI 19‐43) and 9 (95% CI 6‐14), respectively.

### 3.3 Relative risk of hyperglycaemia requiring initiation of new hypoglycaemic therapy after glucocorticoid treatment in all individuals

Seven studies reported the association between glucocorticoids and hyperglycaemia requiring initiation of new hypoglycaemic therapy. The relative risk, comparing glucocorticoid treatment with placebo, was 1.70 [95% CI 1.40‐2.14; p < .001, I^2^ = 0% (Figure 1B)]. The funnel plot suggests evidence of publication bias (Supplementary Figure S3).

### 3.4 Relative risk of hyperglycaemia after glucocorticoid treatment in individuals without known diabetes

**Figure 2:**
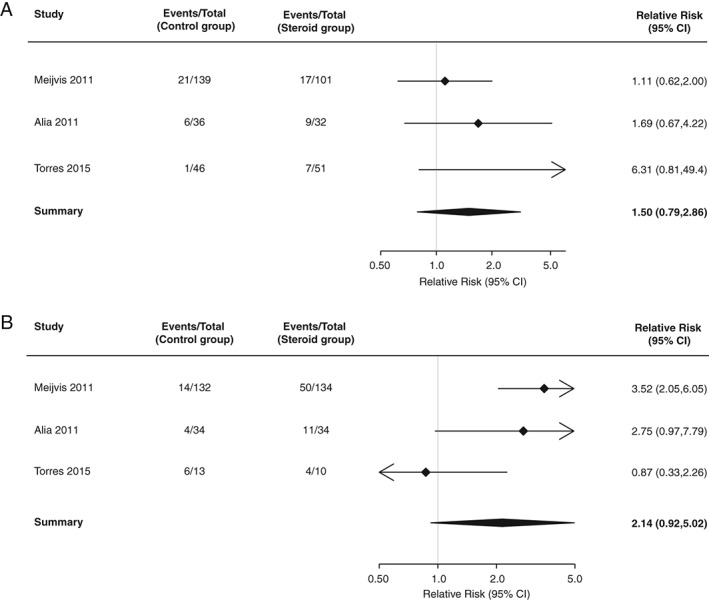
(A) Relative risk of hyperglycaemia, comparing glucocorticoid treatment with placebo in individuals with diabetes at study entry. (B) Relative risk of hyperglycaemia, comparing glucocorticoid treatment with placebo in individuals without diabetes at study entry.

Three studies reported the association between glucocorticoid treatment and hyperglycaemia in individuals without known diabetes.^8^, ^10^, ^12^ The relative risk, comparing glucocorticoid treatment with placebo, was 1.50 [95% CI 0.79‐2.86; p = .22, I^2^ = 29% (Figure 2A)].

### 3.5 Relative risk of hyperglycaemia after glucocorticoid treatment in individuals with known diabetes

Three studies reported the association between glucocorticoid treatment and hyperglycaemia in individuals with known diabetes.^8^, ^10^, ^12^ The relative risk, comparing glucocorticoid treatment with placebo, was 2.10 (95% CI 0.92‐5.02; p = .079, I^2^ = 68% (Figure 2B)].

## 4 DISCUSSION

The findings of this meta‐analysis show that trial participants randomized to glucocorticoid treatment are more likely to develop hyperglycaemia than those randomized to placebo. We found no evidence of a significantly increased risk of hyperglycaemia for individuals analysed based on diabetic status who were randomized to glucocorticoid treatment.

The risk increase we report depends on the definition of hyperglycaemia in the trials. Six of eight studies did not provide a definition of hyperglycaemia. In one study, hyperglycaemia was defined as plasma blood glucose concentration ≥180 mg/dL (10 mmol/L),^6^ whereas a second study defined hyperglycaemia as glucose concentrations ≥120 mg/dL (6.7 mmol/L).^8^ Despite these differences, we found little important heterogeneity between studies. By limiting our analyses to randomized clinical trials, we have minimized the possibility that the patients most likely to develop hyperglycaemia were also those most likely to receive glucocorticoids; however, we cannot exclude the possibility that detection bias occurred. For example, patients randomized to glucocorticoids may have experienced other glucocorticoid‐related adverse effects which caused them to seek medical care where clinicians documented hyperglycaemia. We are also aware, however, that patients randomized to placebo may have been more likely than patients randomized to glucocorticoids to seek medical attention for their underlying respiratory disease; therefore, it is unclear in which direction this potential bias might influence the estimates we report.

A Cochrane review systematically assessed the risk of hyperglycaemia in six studies that administered glucocorticoids to treat acute exacerbations of COPD; however, diabetic status was not ascertained.^13^ Of the studies included, one relied on glycosuria as the measure of glycaemic control, which is not current standard of care. Another study did not include glucose monitoring as part of the study protocol, and therefore may have missed hyperglycaemia. Our estimate is lower (1.72) than the odds ratio (2.8) reported in the Cochrane review estimating the association between glucocorticoid use and hyperglycaemia in a population comprising people with and without diabetes.

Observational studies have estimated odds ratios ranging between 1.36 and 2.31 for the association between glucocorticoid therapy and new‐onset diabetes mellitus; however, the primary endpoints for these studies were new prescriptions filled for glucocorticoids^3^ or hypoglycaemic agents^14^ rather than biochemical assessment of glycaemia. A meta‐analysis found that 19% of patients without diabetes developed glucocorticoid‐induced hyperglycaemia after glucocorticoid treatment^4^; however, the study did not report hyperglycaemia risk in patients with diabetes not treated with glucocorticoids. Moreover, the majority of studies analysed in the meta‐analysis were retrospective, combined a wide range of patient populations and disease states, and, as did our study, identified publication bias.

Glucocorticoids increase hepatic gluconeogenesis, reduce peripheral glucose uptake, inhibit insulin secretion and cause postprandial hyperglycaemia. Seven studies included in the present analysis did not define the timing of blood glucose measurements. One study, in which patients were randomized to once‐daily intravenous dexamethasone or placebo, sampled blood daily at 08:00 hours, which may not be sensitive to hyperglycaemia^10^ and may have biased the effect measure we report. It has been shown that glucocorticoids administered in the morning increase the value of blood glucose into the evening, peaking 8 hours later, diminishing overnight, and resulting in normal fasting glucose levels the following morning.^15^

In summary, the data suggest that glucocorticoids increase the risk of hyperglycaemia in patients with respiratory disease. In subjects analysed based on diabetic status alone, if an association between treatment with glucocorticoids and hyperglycaemia exists, then this study did not find it. Our estimates may reflect publication bias. We recommend that future trials of glucocorticoids determine glycaemia at baseline, during follow‐up, and at time intervals that reflect the pharmacokinetic actions of glucocorticoids.

## Financial Disclosure

S. J. S. is supported by the Medical Research Council (Grant: MC_UU_12015/1).

## Author contributions

B.G.C and A.A designed the study. S.B and B.G.C collected the data. S.S performed statistical analysis. B.G.C and A.A wrote the manuscript.

## Supporting information


**Figure** **S** **1.** Study flow diagram illustrating selection process for published studies included in the final meta‐analysis.

**Figure** **S** **2.** Funnel plot to assess for small studies effect in the analysis of the relative risk of hyperglycaemia comparing glucocorticoid treatment with placebo in all individuals. Studies above the dark contour have p < .01, studies between the light and dark contour have .01 < p < .05, studies below the light contour have p > .05.

**Figure** **S** **3.** Funnel plot to assess for small studies effect in the analysis of relative risk of hyperglycaemia requiring initiation of hypoglycaemic therapy comparing glucocorticoid treatment with placebo in all individuals. Studies above the dark contour have p < .01, studies between the light and dark contour have .01 < p <.05, studies below the light contour have p > .05.

**Table** **S** **1.** Baseline characteristics of participants in the seven studies included in the meta‐analysis. N/A, not available.
