# Supplementary material for: Glucocorticoid‐induced hyperglycaemia in respiratory disease: a systematic review and meta‐analysis
Source: Diabetes Obes Metab. 2016 Aug 4;18(12):1274–8. doi: 10.1111/dom.12739 (PMC5111607; doi:10.1111/dom.12739)
Supplement: Supplementary file 1 — Figure S 1. Study flow diagram illustrating selection process for published studies included in the final meta‐analysis. [file DOM-18-1274-s002.pptx]

## Slide 1
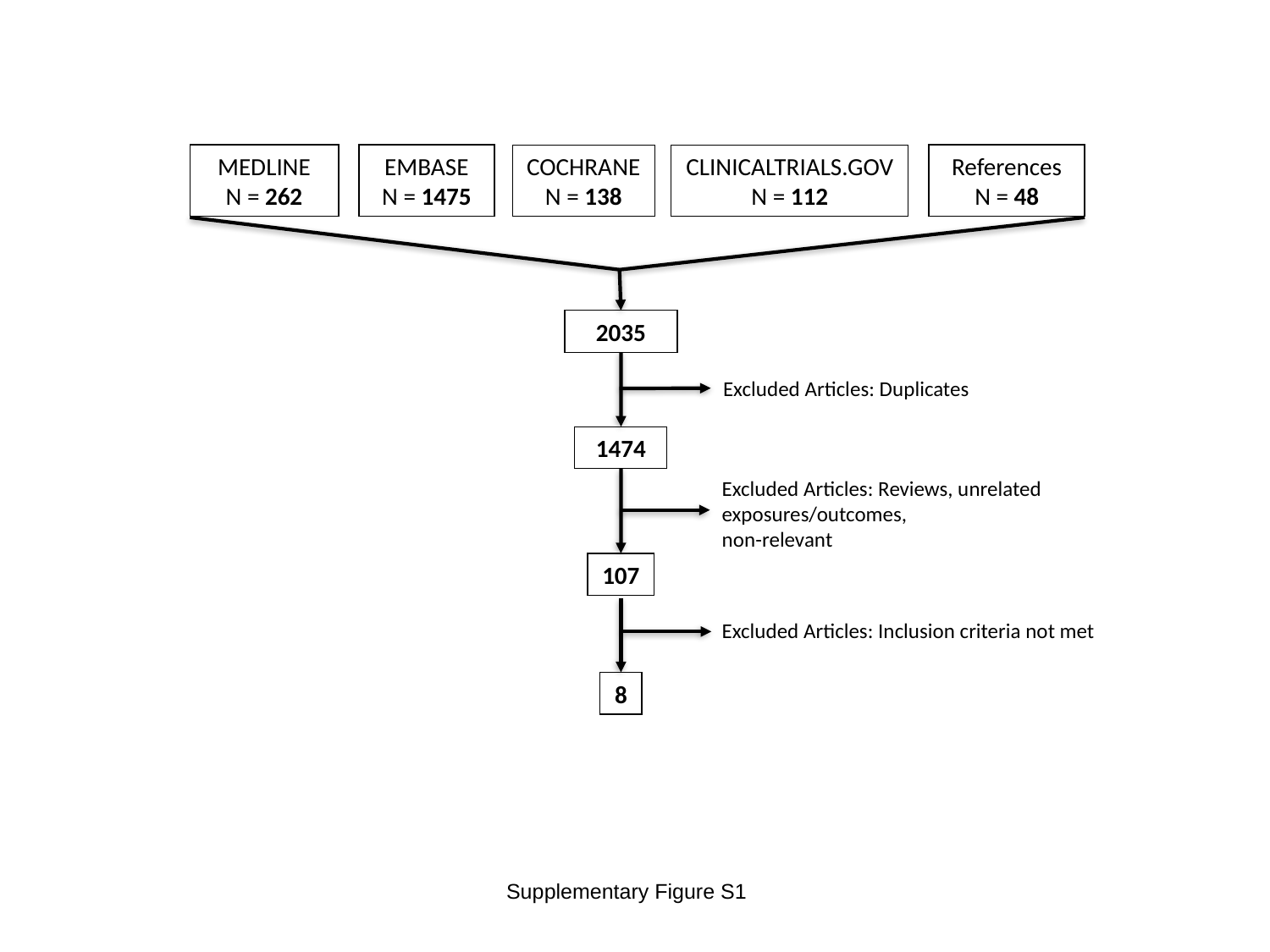

MEDLINE
N = 262
EMBASE
N = 1475
COCHRANE
N = 138
CLINICALTRIALS.GOV
N = 112
References
N = 48
2035
Excluded Articles: Duplicates
1474
Excluded Articles: Reviews, unrelated exposures/outcomes,
non-relevant
107
Excluded Articles: Inclusion criteria not met
8
Supplementary Figure S1
