# Supplementary figures and images for: Glucocorticoid‐induced hyperglycaemia in respiratory disease: a systematic review and meta‐analysis
Source: Diabetes Obes Metab. 2016 Aug 4;18(12):1274–8. doi: 10.1111/dom.12739 (PMC5111607; doi:10.1111/dom.12739)

## Slide 1
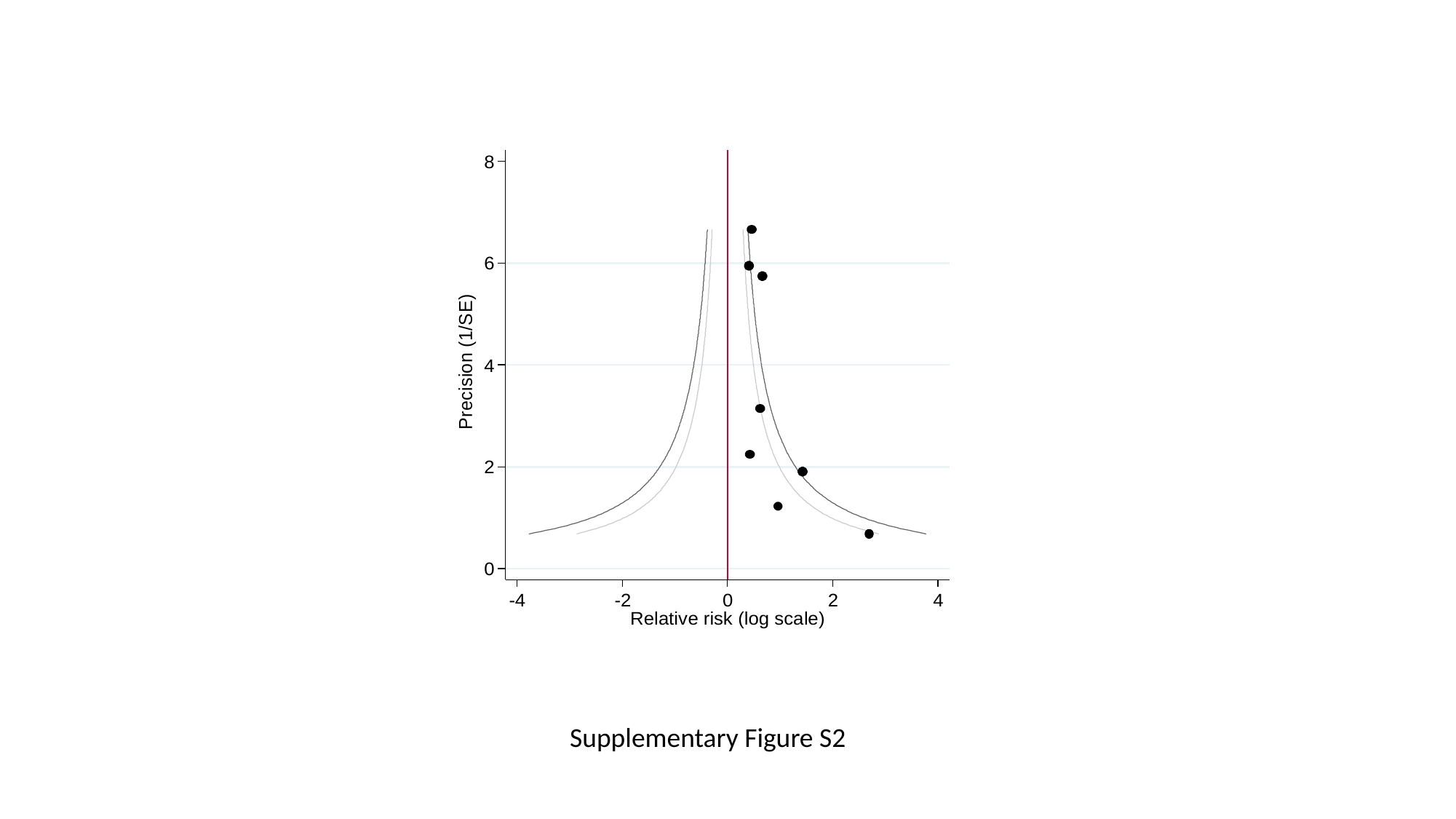

Supplementary Figure S2

Supplement: Supplementary file 2 — Figure S 2. Funnel plot to assess for small studies effect in the analysis of the relative risk of hyperglycaemia comparing glucocorticoid treatment with placebo in all individuals. Studies above the dark contour have p < .01, studies between the light and dark contour have .01 < p < .05, studies below the light contour have p > .05. [file DOM-18-1274-s001.pptx]

## Slide 1
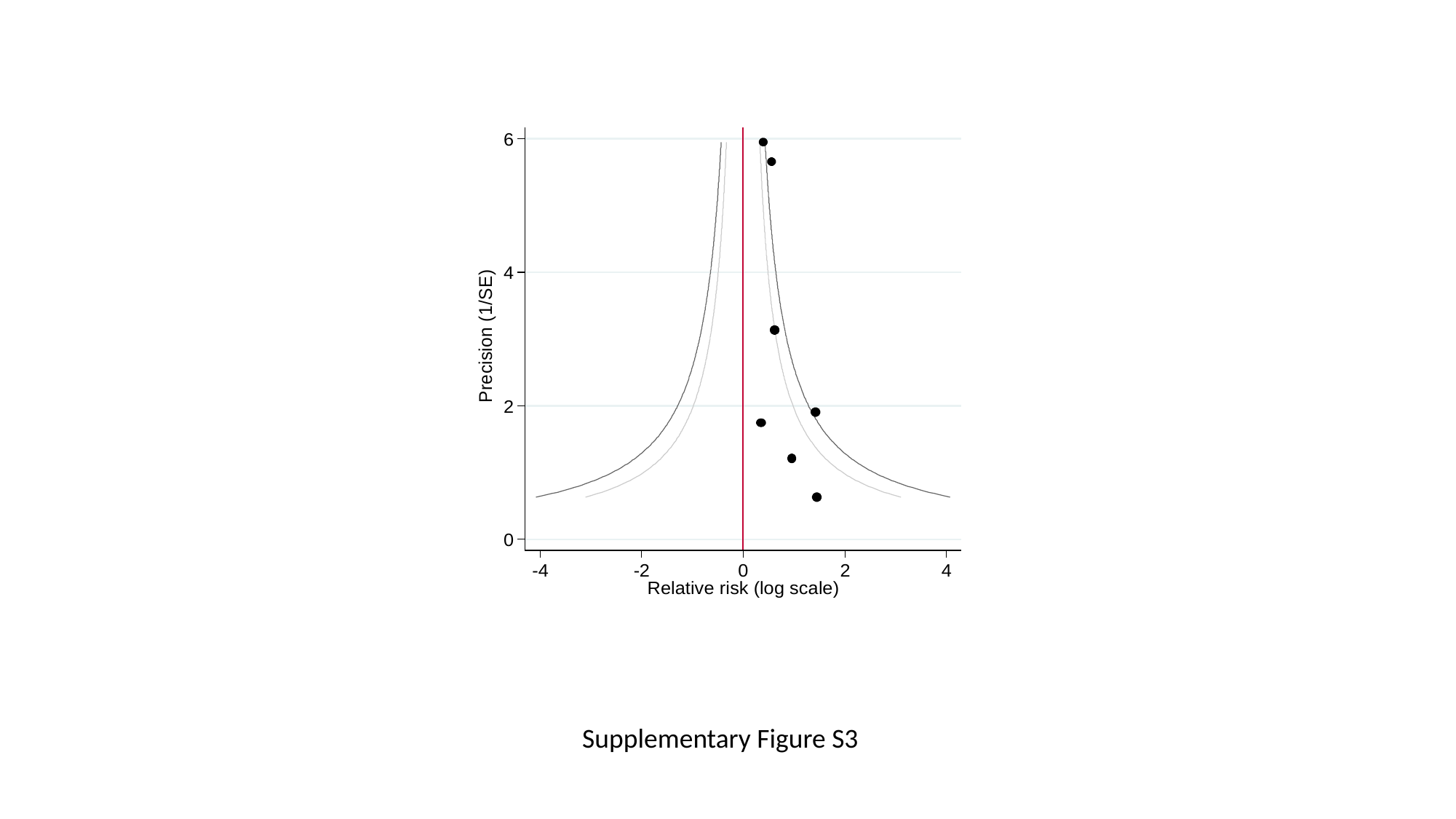

Supplementary Figure S3

Supplement: Supplementary file 3 — Figure S 3. Funnel plot to assess for small studies effect in the analysis of relative risk of hyperglycaemia requiring initiation of hypoglycaemic therapy comparing glucocorticoid treatment with placebo in all individuals. Studies above the dark contour have p < .01, studies between the light and dark contour have .01 < p <.05, studies below the light contour have p > .05. [file DOM-18-1274-s003.pptx]
