# Supplementary material for: Glucocorticoid‐induced hyperglycaemia in respiratory disease: a systematic review and meta‐analysis
Source: Diabetes Obes Metab. 2016 Aug 4;18(12):1274–8. doi: 10.1111/dom.12739 (PMC5111607; doi:10.1111/dom.12739)
Supplement: Supplementary file 4 — Table S 1. Baseline characteristics of participants in the seven studies included in the meta‐analysis. N/A, not available. [file DOM-18-1274-s004.docx]

| **First author, year** | ***Niewoehner, 1999*** | ***Maltais, 2002*** | | ***Snijders, 2010*** | ***Meijvis, 2011*** | | ***Alia, 2011*** | | ***Abroug, 2014*** | ***Blum, 2015*** | ***Torres, 2015*** |
| --- | --- | --- | --- | --- | --- | --- | --- | --- | --- | --- | --- |
|  |  |  | |  |  | |  | |  |  |  |
| **Primary outcome** | Treatment failure | Change in postbronchodilator FEV_1_ | | Clinical outcome (Day 7) | Length of hospital stay | | Duration of mechanical ventilation, length of ICU stay & need for intubation | | Intensive care unit mortality | Time to clinical stability | Treatment Failure |
| **Trial design** | Randomised, double-blind, placebo-controlled trial | Randomised, double-blind, placebo-controlled trial | | Randomised, double-blind placebo controlled trial | Randomised, double-blind placebo-controlled trial | | Randomised, double- blind, placebo-controlled trial | | Randomised, single blind (subject) efficacy trial | Randomised, double-blind, placebo controlled trial | Randomised, double-blind, placebo-controlled trial |
| **Study location** | USA | Belgium, Canada & France | | Netherlands | Netherlands | | Spain | | Tunisia | Switzerland | Spain |
| **Baseline year** | 1994-1996 | N/A | | 2005-2008 | 2007-2010 | | 2005-2009 | | 2010-2011 | 2009-2014 | 2004-2012 |
| **Subjects, n** | 271 | 128 | | 213 | 304 | | 83 | | 217 | 785 | 120 |
| **Males, %** | 98.9 | 82 | | 58.2 | 56.3 | | 79.5 | | 88 | 62 | 62 |
| **Mean age, years** | 67.7 | 70.4 | | 63.5 | 63.6 | | 68.4 | | N/A | N/A | 65.3 |
| **Respiratory illness** | COPD Exacerbation | COPD Exacerbation | | Community acquired pneumonia | Community acquired pneumonia | | COPD Exacerbation | | COPD Exacerbation | Community acquired pneumonia | Community acquired pneumonia |
| **Diabetes mellitus at baseline, n (%)** | 10.3 | N/A | | 10.3 | 14.1 | | 28.9 | | 13.8 | 19.7 | 19.2 |
|  |  |  | |  |  | |  | |  |  |  |
| **Glucocorticoid regime** | IV methylprednisolone (125mg every 6hrs for 72hrs) followed by tapering schedule oral prednisone for 8 weeks or 12 days followed by 6 weeks placebo. | Oral prednisolone (30mg every 12 hours) for 72h followed by prednisolone (40mg daily) plus for 7 days | | IV/ oral prednisolone (40mg daily) for 7 days | IV dexamethasone (5mg daily) within 12 hours of admission plus 3 days. | | IV methylprednisolone 0.5 mg/kg every 6 hours for 72 hours, 0.5 mg/kg every 12 hours on days 4 through 6, 0.5 mg/kg daily for day 7 through day 10 | | Oral prednisone (1 mg/kg daily) until discharge or maximum 10 days | Oral prednisone (50 mg daily) for 7 days | IV methylprednisolone (0.5mg/kg) every 12 hours for 5 days. |
|  |  |  | |  |  | |  | |  |  |  |
| **Definition of hyperglycaemia** | Initiation of insulin or oral hypoglycaemic agent in subjects without diabetes or increased doses of glucose lowering therapies in subjects with diabetes mellitus | N/A | | N/A | Non-fasting plasma glucose > 11mmol/l | | Blood glucose >120 mg/dL resulting in initiation of insulin therapy in subjects without diabetes mellitus or intensified insulin therapy in subjects with diabetes mellitus. | | Blood glucose >180mg/dl necessitating initiation of insulin therapy in subjects without diabetes or intensified insulin therapy in subjects with diabetes. | New onset hyperglycaemia requiring new insulin treatment. | N/A |
| **Blood glucose monitoring regime** | N/A | Plasma blood glucose measured at trial entry and at 72 hours. | | Plasma blood glucose measured at baseline & daily for 7 days. | Plasma blood glucose measured at presentation, 0800h on study days 1-7 and 30 days after study entry. | | Blood glucose measured on study days 1-5. | | N/A | Four times daily glucose measurements on study days 1,3,5,7 and discharge. | N/A |
| **Length of follow-up** | 6 months | 10 days | 30 days | | | 30 days | | Length of ICU stay or mortality | Length of ICU stay or mortality | Time to clinical stability or hospital discharge | 120 hours |

**Supplementary Table S1**
